# Splenic Infarct in a Patient with Autoimmune Hemolytic Anemia

**DOI:** 10.5505/tjh.2012.71324

**Published:** 2012-12-05

**Authors:** Hava Üsküdar Teke, Samet Karahan, Ümmügülsüm Gümüş

**Affiliations:** 1 Kayseri Education and Research Hospital, Department of Hematology, Kayseri, Turkey; 2 Kayseri Education and Research Hospital, Department of Internal Medicine, Kayseri, Turkey; 3 Kayseri Education and Research Hospital, Department of Radiology, Kayseri, Turkey

**To the Editor,**

Splenic infarct may be observed during the course of several diseases; however, the incidence is quite low. Splenic infarct is related to thromboembolic events; various diseases, including sickle cell anemia, chronic myeloproliferative diseases, lymphoma, and leukemia, can also cause splenic infarct [1]. In patients with autoimmune hemolytic anemia (AIHA) massive hemolysis causes activation of the immune response, destruction of erythrocytes, and splenomegaly [2]. Splenic infarct is rarely observed in AIHA patients and to the best of our knowledge the literature contains only 3 cases of splenic infarct due to AIHA [3,4,5]. Herein we describe a 67-year-old female that presented to the emergency department with fatigue, tachycardia, and weakness. 

The patient’s vital signs were as follows: temperature: 37 °C; pulse: 120/min; blood pressure: 120/70 mmHg. Physical examination showed pale conjunctivas, icteric sclerae, and a spleen that was palpable 3 cm below the costa. Lymphadenopathy was not observed. Electrocardiography (ECG) and lung radiography results were normal. Complete blood count findings were as follows: Hb: 5.7 g/dL; MCV: 115 fL, WBC count: 6.4 x 10^9^/L; Plt count: 157 x 10^9^/L. Biochemical examination findings were as follows: total bilirubin: 2.0 mg/dL; indirect bilirubin: 1.5 mg/dL; LDH: 466 U/L. Her reticulocyte count was 6.38%, direct anti-globulin test with IgG and C3d complement was 4 positive, and the indirect anti-globulin test was 4positive. Erythrocyte agglutination, polychromasia, and spherocytes were observed in her peripheral blood smear. 

The patient was further examined to determine if there existed other secondary causes for her complaints; there were no signs of hepatitis, her IgG, IgA, and IgM levels were normal, and monoclonality was not observed on the serum protein electrophoresis. Mycoplasma pneumonia, ANA, anti-ds DNA, and HIV tests were negative. Lymphoma was eliminated via computed tomography (CT). Bone marrow aspiration showed the presence of erythroid hyperplasia. Based on these findings, the patient was diagnosed with AIHA. The patient was given erythrocyte suspension because of her symptomatic status. The patient was started on 1 mg/kg/d methylprednisolone and folic acid after the diagnosis of idiopathic mixed type AIHA. 

After 3 weeks of the steroid treatment, the patient developed left upper quadrant pain and and the abdominal ultrasonography revealed splenic infarct complicated by splenic hemorrhage. Upper abdominal MRI was performed to confirm the presence of splenic infarct. MRI showed that the spleen was 155 mm and that a wedge-shaped hemorrhagic infarct was present around the spleen (Figure). The patient was experiencing severe pain in the upper left quadrant and underwent splenectomy, a common treatment for AIHA. The patient’s pain disappeared following the splenectomy. To eliminate hypercoagulability causes protein C, protein S, antithrombin, factor VIII, factor V Leiden mutation, prothrombin mutation, lupus anticoagu-lant, anti-cardiolipin antibodies, and homocysteine were measured, all of which were normal. ECG showed a normal sinus rhythm. Transthoracic echocardiography did not show any cardiac valve pathology. Thromboemboli were not present during the observation. The patient was started on azathioprine for AIHA, as she was unresponsive to steroid treatment and splenectomy. 

The most common cause of splenic infarct is cardiovascular thromboembolism (22% of all cases), followed by thrombophilia[5]. In addition, the third most common cause of splenic infarct is hematological diseases, including sickle hemoglobinopathies, myelofibrosis, paroxysmal nocturnal hemoglobinuria, polycythemia vera, leukemia, and lymphoma, which account for 10% of all cases [1]; however, only 3 cases of splenic infarct due to AIHA have been reported [3,4,5]. 

Thrombogenic events and splenic vasculitis are not the only causes of splenic infarct. Other immune-mediated mechanisms related to cryoglobulin synthesis can cause splenic infarct [6]. In cases of cardiovascular thromboembolism or thrombophilia-induced infarct, the lungs, kidneys, heart, and intestine, together with splenic infarct, are affected by multiple emboli; however, in hematological diseases infarct is usually limited to the spleen [5]. The presented patient was evaluated for thrombophilia, but there was neither a laboratory evidence for hypercoagulability nor any cardiac cause. Moreover, the presented case only had an infarct that was localized in the spleen. Splenic infarct may have arisen in the present case due to splenomegaly or immune-mediated mechanisms caused by cold antibodies; the patient had both warm and cold antibodies. 

Medical treatment can be administered for uncomplicated splenic infarcts. Splenectomy is preferred in cases with persistent symptoms or complicated cases with a splenic pseudocyst, abscess, or hemorrhage; however, early splenectomy is required to prevent mortality [7]. The presented case had pain that was unresponsive to analgesic treatment, and her splenic infarct was complicated by the presence of a hemorrhage; therefore, splenectomy was performed with early intervention. Following the splenectomy the patient’s complaints disappeared. 

In conclusion, in patients with such hematological diseases as AIHA, hemoglobinopathies, polycythemia vera, myelofibrosis, leukemia, and lymphoma, and complaints of left upper quadrant pain, splenic infarct should be suspected and MRI or CT should be performed for diagnostic purposes. As thrombophilia can also cause infarct, patients should also be evaluated for thrombophilia. If a patient with splenic infarct complains of severe persistent pain and the case is complicated the patient should undergo splenectomy immediately to avoid mortality. 

**Conflict of Interest Statement**

None of the authors have any conflicts of interest, including specific financial interests, relationships, and/or affiliations, relevant to the subject matter or materials included.

## Figures and Tables

**Figure:**
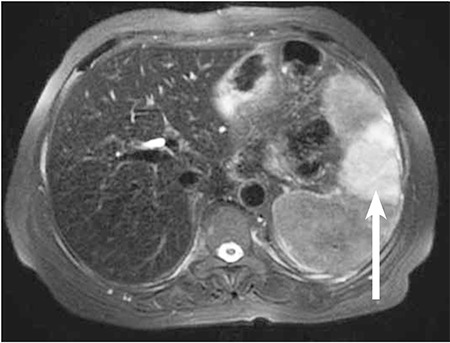
Axial T2-weighted fat-suppressed MRI shows a wedge shaped hyperintense area of infarction in the spleen (arrow), as well as decreased signal intensity of the liver
